# Drug use in delivery hospitalization: Pelotas births cohort, 2015

**DOI:** 10.11606/S1518-8787.2019053000913

**Published:** 2019-05-21

**Authors:** Marysabel Pinto Telis Silveira, Vanessa Iribarrem Avena Miranda, Mariângela Freitas da Silveira, Tatiane da Silva Dal Pizzol, Sotero Serrate Mengue, Andréa Dâmaso Bertoldi

**Affiliations:** I Universidade Federal de Pelotas. Departamento de Fisiologia e Farmacologia. Instituto de Biologia. Pelotas, RS, Brasil; II Universidade Federal de Pelotas. Programa de Pós-Graduação em Epidemiologia. Pelotas, RS, Brasil; III Universidade Federal de Pelotas. Departamento Materno-Infantil. Pelotas, RS, Brasil; IV Universidade Federal do Rio Grande do Sul. Departamento de Produção e Controle de Medicamentos. Faculdade de Farmácia. Porto Alegre, RS, Brasil; V Universidade Federal de Pelotas. Departamento de Medicina Social. Pelotas, RS, Brasil

**Keywords:** Parturition, Midwifery, Perinatal Care, Drug Utilization, Risk Factors, Socioeconomic Factors, Parto, Tocologia, Assistência Perinatal, Uso de Medicamentos, Fatores de Risco, Fatores Socioeconômicos

## Abstract

**OBJECTIVE:**

Trace the pattern of drug use during delivery hospitalization.

**METHOD:**

Cross-sectional study carried out from June to October 2015, included in the 2015 Pelotas births cohort. All women living in the urban area of the city who were hospitalized for delivery were part of the sample. We collected information regarding drug prescription and drug use by mothers during the whole period of hospitalization. Sociodemographic data were obtained in interview after delivery, and other data were obtained from medical charts. The drugs were classified according to the Anatomical Therapeutic Chemical system.

**RESULTS:**

All study participants (1,392 women) used at least one drug, with the mean amount being larger the higher the age of the mother, both prepartum/during delivery and postpartum. It was also higher in cases of spinal anesthesia or general anesthesia, cesarean deliveries, school hospitals, and longer hospitalizations. Analysis of the sample as a whole showed no significant difference in the number of drugs used according to hospitalization type, but when stratified by length of hospital stay the mean was higher in SUS hospitalizations than in private and health insurance hospitalizations. Drugs for the nervous system were the most used (30.5%), followed by drugs for the alimentary tract and metabolism (13.8%). The use of anti-infective agents and drugs that act on the cardiovascular and respiratory systems was higher in mothers who underwent cesarean delivery. This study showed high drug consumption in the delivery hospitalization period, and showed cesarean delivery and epidural anesthesia as the main factors related to high drug consumption in this period.

**CONCLUSIONS:**

We found high drug consumption in the delivery hospitalization period, and the main factors were cesarean delivery and epidural anesthesia. Drugs that act on the nervous system were the most used.

## RESUMO

**OBJETIVO:**

Identificar o padrão de uso dos medicamentos durante a internação para o parto.

**MÉTODO:**

Estudo transversal realizado de junho a outubro de 2015, inserido na coorte de nascimentos de Pelotas de 2015. Todas as mulheres residentes na zona urbana da cidade que foram internadas para o parto fizeram parte da amostra. Foram coletadas informações referentes à prescrição e uso de medicamentos pela mãe durante todo o período de internação. Dados sociodemográficos foram obtidos na entrevista realizada após o parto, e os demais nos prontuários. Os medicamentos foram classificados de acordo com o sistema *Anatomical Therapeutic Chemical*.

**RESULTADOS:**

Todas as participantes do estudo (1.392 mulheres) utilizaram pelo menos um medicamento, sendo a quantidade média maior quanto maior a idade da mãe, tanto no momento pré/durante o parto como no pós-parto. Também foi maior em casos de raquianestesia ou anestesia geral, partos cesarianos, hospitais escola e internações mais prolongadas. Na análise da amostra como um todo, não houve diferença significativa no número de medicamentos utilizados de acordo com o tipo de hospitalização, mas quando estratificada por período de internação, a média foi maior nas internações pelo SUS que nas internações particulares e por convênios. Medicamentos para o sistema nervoso foram os mais utilizados (30,5%), seguidos dos que atuam no trato alimentar e metabolismo (13,8%). O uso de anti-infecciosos e fármacos que atuam nos sistemas cardiovascular e respiratório foi maior em mães que fizeram cesariana. Este estudo evidenciou elevado consumo de medicamentos no período de internação para o parto, e o parto cesariano e a anestesia peridural como os principais fatores relacionados ao consumo elevado de medicamentos neste período.

**CONCLUSÕES:**

Evidenciou-se elevado consumo de medicamentos no período de internação para o parto, sendo os principais fatores a cesariana e a anestesia peridural. Os medicamentos que atuam no sistema nervoso foram os mais utilizados.

## INTRODUCTION

Drug use in hospitals is highly prevalent, independently of health problem or hospital sector. During delivery hospitalization, drugs are used to induce labor, reduce pain, or treat preexisting maternal diseases that require maintenance of pharmacological control. The use of these drugs, although clinically justified, may affect the health of the newborn. In all cases, the choice of drug should include evaluation of safety of use during the gestation period^1^ ^,^ ^2^.

In recent years some researches were conducted in developed countries using large databases or national administrative records, which have been increasingly used in the field of perinatal pharmacoepidemiology to attempt to address the deficiency of clinical epidemiological studies on pregnant women^3^ ^–^ ^7^. However, Brazil lacks pharmacoepidemiological databases to conduct studies with such objective.

The characterization of drug prescription during delivery hospitalization has been little investigated, in comparison with drug use throughout gestation^8^ ^,^ ^9^. This difference may be explained, in part, by the higher importance attributed to the teratogenic risks of drugs^8^. A study conducted in a maternity hospital of Minas Gerais analyzed all drug prescriptions during one year, but comprising prescriptions of all hospitalizations of the maternity hospital and not only those of women admitted for delivery^10^. Another study conducted in a maternity hospital of Ceará evaluated drug use in the immediate postpartum period and 15 days after hospital discharge, but not the entire delivery hospitalization period^11^.

Drug use in this period may also have undesired effects on the newborn, such as respiratory depression^9^ ^,^ ^12^. In addition, analysis of drug use between different health care services (for example, between public and private maternity hospitals) may show differences in therapeutic conducts. Investigating the improper use of certain drugs, such as the indiscriminate use of oxytocin in labor, may show irrational practices in the employment of drugs^13^.

This study aimed to characterize the pattern of drug use during delivery hospitalization.

## METHODS

Cross-sectional study carried out from June 5 to October 5, 2015, included in the 2015 Pelotas births cohort (C2015). The sample comprised all women living in the urban area of the city of Pelotas, state of Rio Grande do Sul, colônia Z3 and Jardim América neighborhood of the municipality of Capão do Leão who were hospitalized for delivery in the five hospitals of the city in the study period, regardless of whether the childbirth resulted in stillborn or born alive. More details on the perinatal study of C2015 can be found in the methodological article^14^.

We collected information regarding drug prescription and drug use by mothers during the whole hospitalization period. We obtained sociodemographic data of the mothers in interview conducted shortly after childbirth, still in hospital, by previously trained interviewers of the cohort. We analyzed the following variables: age (collected in full years and categorized into the 13–19, 20–30, and 31–45 ranges), economic classification according to the criteria of the Brazilian Association of Survey Companies^15^ (A, B, C, or D+E); self-reported skin color (white, black, or brown – which includes dark, brown, and indigenous), and educational level (collected in years of continuous study and categorized into the 0–4, 5–8, 9–11, or ≥ 12 ranges).

The other data were collected from medical charts, soon after hospital discharge, by previously trained Pharmacy students and nursing technicians. We registered the following information: hospital of birth, anesthesia type (spinal, epidural, topical, general, or none), delivery type (vaginal or caesarian delivery), and hospitalization type (Unified Health System – SUS, health insurance plan, or private health care). The variables for hospitalization days, hospitalization days before delivery, and hospitalization days after delivery were based on the dates of admission, delivery, and discharge.

As for the drugs used, we obtained information on the drug name, time of use (prepartum/during delivery or postpartum), and days of use. We used the International classification *Anatomical Therapeutic Chemical* (ATC) of the World Health Organization^16^ to classify them by therapeutic groups to the fifth level, when possible. In order not to identify the hospitals, they were named with the letters from A to E, with A and C being medical school hospitals.

The information was typed directly on a tablet and later transferred to the computer, and the data were analyzed with the statistical package Stata version 12.0 (Stata Corp., College Station, USA). In the descriptive analysis, we used the analysis of variance (ANOVA) to evaluate possible associations between the mean and standard deviation (SD) of the number of drugs used in each category of exposure variables (mothers being analysis units). To analyze the distribution of the most used drugs according to therapeutic group and delivery type, anesthesia type, mother age, and birth hospital, we used the bank in the long format, considering the drug as analysis unit. For these analyses we estimated the prevalence and the 95% confidence intervals.

The project was approved by the Research Ethics Committee of the Medical School of the Federal University of Pelotas, under number 315,264, including only pregnant women who agreed to participate in the study and signed the informed consent form.

## RESULTS

1,392 women participated in the study of the 4,270 who were part of the 2015 perinatal cohort study. The mean age was 27 years (SD = 6.7; minimum = 13; maximum = 45). Most of the sample (65.9%) had nine or more years of formal education and belonged to economic class C (48.7%). Only five declared being indigenous, and the same number as having yellow skin color, being grouped with those who declared having dark or brown skin (13.2%); 70.0% declared having white skin color (Table 1). The distribution of this sample, in relation to the sociodemographic variables presented, was similar to the total of the cohort (p > 0.05). Medical school hospitals totaled more than 50% of deliveries.

**Table 1:** Description of sample and mean number of drugs used during delivery hospitalization according to sociodemographic variables and hospitalization characteristics. 2015 Pelotas Births Cohort (June to October), Brazil.

Variable		Sample	Mean number of drugs^a^		
		n (%)	All	Prepartum/during delivery	Postpartum
			Mean (SD)	Mean (SD)	Mean (SD)
Age (years)			p = 0.0004	p ≤ 0.001	p = 0.041
	13–19	211 (15.2)	9.98 (4.9)	6.5 (4.3)	5.3 (2.4)
	20–30	742 (53.3)	10.1 (4.4)	6.7 (3.9)	5.4 (2.2)
	31–45	439 (31.6)	11.0 (4.3)	7.6 (3.9)	5.7 (2.3)
Skin color^b^			p = 0.643	p = 0.078	p = 0.230
	White	975 (70.0)	10.4 (4.3)	7.0 (3.8)	5.4 (2.1)
	Black	232 (16.7)	10.1 (4.9)	6.4 (4.3)	5.7 (2.5)
	Brown^c^	183 (13.2)	10.4 (4.9)	7.0 (4.3)	5.6 (2.6)
Educational level of mother (complete years of formal education)			p = 0.243	p = 0.011	p = 0.106
	0–4	119 (8.6)	9.6 (5.6)	6.3 (5.0)	5.4 (2.3)
	5–8	355 (25.4)	10.2 (4.7)	6.6 (4.2)	5.7 (2.3)
	9–11	472 (33.9)	10.4 (4.6)	6.9 (4.2)	5.6 (2.3)
	12 or more	446 (32.0)	10.5 (3.7)	7.4 (3.1)	5.3 (2.1)
Economy class (ABEP)^d^			p = 0.485	p = 0.202	p = 0.08
	A	51 (3.8)	11.2 (2.9)	7.8 (2.5)	5.7 (2.1)
	B	375 (27.9)	10.3 (3.4)	7.1 (3.0)	5.3 (2.1)
	C	654 (48.7)	10.3 (4.7)	6.8 (4.1)	5.6 (2.3)
	D+E	262 (19.5)	10.2 (5.2)	6.8 (4.8)	5.5 (2.3)
Hospital of birth			p ≤ 0.001	p ≤ 0.001	p ≤ 0.001
	A	514 (36.9)	10.3 (4.1)	6.5 (3.4)	5.7 (2.2)
	B	496 (35.6)	9.7 (3.8)	6.2 (3.2)	5.5 (2.1)
	C	243 (17.5)	12.0 (6.5)	8.8 (6.1)	6.0 (2.6)
	D	126 (9.1)	9.8 (2.4)	7.9 (2.2)	3.9 (1.2)
	E	14 (1.0)	9.7 (3.3)	7.7 (2.3)	4.4 (2.3)
Type of anesthesia			p ≤ 0.001	p ≤ 0.001	p ≤ 0.001
	Spinal anesthesia^e^	929 (66.7)	12.6 (3.5)	8.8 (3.2)	6.3 (2.2)
	Topical anesthesia	341 (24.5)	5.9 (1.9)	3.2 (1.6)	3.9 (1.4)
	General anesthesia	4 (0.3)	13.3 (7.1)	11.3 (6.8)	3.0 (2.2)
	None	118 (8.5)	5.6 (2.7)	2.7 (2.6)	4.0 (1.7)
Type of delivery			p ≤ 0.001	p ≤ 0.001	p ≤ 0.001
	Vaginal delivery	462 (33.2)	5.8 (2.2)	3.1 (2.0)	4.0 (1.5)
	Cesarean delivery	930 (66.8)	12.6 (3.6)	8.8 (3.2)	6.3 (2.1)
Type of hospitalization^f^			p = 0.927	p = 0.002	p ≤ 0.001
	Unified Health System (SUS)	884 (66.4)	10.3 (5.0)	6.6 (4.5)	5.6 (2.3)
	Health insurance plan	304 (22.8)	10.3 (2.8)	7.5 (2.5)	5.0 (1.9)
	Private health care	143 (10.7)	10.4 (2.6)	7.2 (2.3)	5.3 (1.8)
Days of hospitalization			p ≤ 0.001	p ≤ 0.001	p ≤ 0.001
	Up to 1	217 (15.6)	9.4 (2.8)	7.1 (2.6)	4.4 (1.7)
	2	489 (35.1)	9.5 (3.6)	6.2 (3.1)	5.2 (1.9)
	3	313 (22.4)	9.4 (4.0)	5.9 (3.5)	5.4 (1.9)
	4 to 10	315 (22.6)	12.1 (5.5)	7.9 (4.9)	6.5 (2.6)
	11 or more	58 (4.2)	16.0 (5.5)	12.7 (4.9)	7.3 (3.2)
Days of hospitalization before delivery			p ≤ 0.001	p ≤ 0.001	p ≤ 0.001
	Up to 1	1.243 (89.3)	9.8 (4.1)	6.4 (3.5)	5.3 (2.1)
	2	46 (3.3)	12.8 (6.0)	9.2 (5.0)	6.5 (2.9)
	3	25 (1.8)	14.1 (4.4)	10.2 (4.0)	6.8 (2.0)
	4 to 10	53 (3.8)	15.9 (4.4)	12.2 (4.1)	7.7 (2.4)
	11 or more	25 (1.8)	17.0 (4.7)	14.1 (4.5)	7.1 (2.7)
Days of hospitalization after delivery			p ≤ 0.001	p ≤ 0.001	p ≤ 0.001
	Up to 1	229 (16.5)	9.3 (2.8)	7.0 (2.6)	4.3 (1.7)
	2	644 (46.3)	9.8 (3.9)	6.5 (3.5)	5.3 (1.9)
	3	277 (19.9)	10.5 (4.4)	6.7 (4.0)	5.9 (2.0)
	4 to 10	229 (16.5)	12.5 (6.1)	8.3 (5.6)	6.7 (2.9)
	11 or more	13 (0.9)	13.9 (5.9)	9.4 (4.2)	7.6 (4.4)
Total		1,392 (100)	10.3 (4.5)	6.9 (4.0)	5.5 (2.2)

a Analysis of variance (ANOVA).

b 2 missing (two mothers did not respond).

c Includes skin colors dark, brown and indigenous.

d Brazilian Association of Survey Companies (52 missing).

e Includes a case of epidural anesthesia.

f 51 missing (information about type of hospitalization was not found in the hospital records).

The most used anesthesia was spinal anesthesia (66.7%), classification into which a woman who received epidural anesthesia was also grouped. General anesthesia was used in four mothers (0.3%) due to complications during delivery. The predominant delivery type was caesarean delivery (66.8%), as shown in Table 1. Only 2 of the 462 women who had vaginal delivery were given spinal anesthesia; 339 women were given topical anesthesia; and 118 were given none.

Analysis of the association between hospital and delivery type shows statistically significant difference, especially as to the hospital that exclusively serves the SUS and to the two hospitals that exclusively serve private health care and health insurance. The other two hospitals are mixed, serving both the SUS and private health care and health insurance. The exclusively SUS hospital held 22.7% (95%CI 18.9–26.6) of all vaginal deliveries and 14.8% (95%CI 12.5–17.1) of caesarean deliveries. The exclusively private hospitals held 2.0% (95%CI 0.7–3.2) of vaginal deliveries and 14.1% (95%CI 11.8–16.3) of cesarean deliveries, while mixed hospitals held 42.2% (95%CI 37.7–46.7) and 33.1% (95%CI 28.8–37.4) vaginal deliveries and 32.3% (95%CI 29.2–35.2) and 38.8% (95%CI 35.6–42.0) of caesarean deliveries.

Mean hospitalization time was 3.6 days (SD=4.0), median of two days (interquartile range – IQR_25-75_ 2–4). After delivery, mean hospitalization time was 2.7 days (SD = 2.2), median of two days (IQR_25-75_ 2–4). We found no statistically significant difference in mean hospitalization days according to delivery type (3.6 days for vaginal delivery and 3.5 days for cesarean delivery), but we found statistically significant difference in the association between hospitalization days and hospitalization type (p < 0.001). The extremes of 1-day hospitalization and 4–10-day hospitalization period are especially interesting. For SUS hospitalization, the ratio of women with 1-day hospitalization was only 0.5% (95%CI 0.09–0.8); for private health care and health insurance hospitalization, that ration was 36.4% (95%CI 28.3–44.3) and 48.3% (95%CI 42.7–54.0), respectively. The length of hospital stay was 4–10 days in 33.4% (95%CI 30.2–36.5) of SUS hospitalizations and in only 1.4% (95%CI 0.5–3.3) and 2.9% (95%CI 1.0–4.8) of private health care and health insurance hospitalizations, respectively.

Description of mean and standard deviation of the number of drugs used during delivery hospitalization, according to exposure variables and hospitalization time (prepartum/during delivery or postpartum), is found in Table 1. All women were given at least one drug, and the mean number was significantly higher the higher the age of the mother, both at prepartum/during delivery and postpartum. It was also significantly higher when spinal anesthesia or general anesthesia were used (in this case mainly at prepartum/during delivery), in cesarean deliveries, in medical school hospitals, and the higher the number of hospitalization days. We found no significant difference in the number of drugs used according to hospitalization type (SUS, health insurance, or private health care) when the entire sample was analyzed; however, when stratified by hospitalization period (prepartum/during delivery or postpartum), the mean number of drugs was significantly higher in SUS hospitalizations than in private health care and health insurance hospitalizations. We found no significant difference in the mean number of drugs used according to economic class (Table 1).

A total of 14,383 drugs were used. Table 2 shows the distribution of the most used drugs, classified according to the Anatomical Therapeutic Chemical system (levels 1 and 3). Drugs that act on the nervous system (ATC group N) were the most used (n = 4,274; 30.5%), followed by those that act on the alimentary tract and metabolism (ATC group A; n = 1,932; 13.8%).

**Table 2:** Distribution of the drugs most used in hospitalization for delivery according to the Anatomical Therapeutic Chemical Classification^a^ (ATC; levels 1 and 3). 2015 Pelotas Births Cohort (June to October), Brazil.

Therapeutic groups		n	%
N – Nervous System		4,274	30.5
	N02B – Other analgesics and antipyretics	1,483	10.6
	N01B – Local anesthetics	1,316	9.4
	N02A – Opioids	938	6.7
	N01A – General anesthetics	188	1.3
	N05A – Antipsychotics	152	1.1
	Others	197	1.4
A – Alimentary tract and metabolism		1,932	13.8
	A04A – Antiemetic and antinauseating agents	822	5.9
	A03A – Drugs for functional disorders	633	4.5
	A03F – Propulsive agents	262	1.9
	Others	215	1.5
M – Musculoskeletal system		1,888	13.5
	M01A – Anti-inflammatory and antirheumatic agents	1,871	13.4
	Others	17	0.1
H – Hormonal preparations for systemic use		1,601	11.3
	H01B – Hormones of the posterior lobe of the pituitary	1,357	9.7
	H02A – Corticosteroids for systemic use	219	1.5
	H03A – Thyroidal preparations	25	0.1
J – Anti-infective for systemic use		1,361	9.7
	J01D – Other beta-lactam antibacterials	1,055	7.5
	Others	306	2.2
B – Blood and blood-forming organs		1,009	7.3
	B03A – Preparations containing iron	747	5.4
	Others	262	1.9
C – Cardiovascular System		963	6.9
	C01C – Cardiac stimulants – except glycosides	685	4.9
	Others	278	2.0
R – Respiratory system		371	2.6
	R06A – Antihistamines for systemic use	368	2.6
	Others	3	0.03
D – Dermatologicals agents		347	2.5
	D08A – Antiseptics and disinfectants	153	1.1
	Others	194	1.4
G – Genitourinary system and sex hormones		195	1.4
	G02A – Uterotonics	180	1.3
	Others	15	0.1
Others		70	0.5
Total		14,011^b^	100

a World Health Organization, Collaborating Centre for Drug Statistics Methodology. Guidelines for ATC classification and DDD assignment 2014. Oslo; 2014 [cited 2016 Mar 15]. Available from: whocc.no/atc_ddd_index/

b Of the 14,383 drugs used, 368 were not classified by the ATC system due to absence of reason for use, which may change the classification of the drug.

Table 3 shows the most used drugs that act on the nervous system, stratified by anesthesia type. For mothers given spinal anesthesia, the most frequent were bupivacaine (30.2%), morphine (23.8%), dipyrone/metamizole (18.5%), and paracetamol (12.0%). For mothers given only topical anesthesia (vaginal area), the most used drugs were paracetamol (53.5%) and bupivacaine (40.3%).

**Table 3:** Distribution of the drugs that act on the nervous system^a^ most used in hospitalization for delivery, stratified by type of anesthesia^b^. 2015 Pelotas Births Cohort (June to October), Brazil.

Drug	Spinal anesthesia			Topical anesthesia		
	n	%	95%CI	n	%	95%CI
Bupivacaine	1,122	30.2	28.7–31.7	189	40.3	35.8–4.7
Morphine	887	23.8	22.5–25.2	1	0.2	-0.2–0.6
Dipyrone (metamizole)	686	18.5	17.2–19.7	25	5.3	3.2–7.3
Paracetamol	446	12.0	10.9–13.1	251	53.5	48.9–8.0
Phentanil	150	4.0	3.4–4.6	1	0.2	-0.2–0.6
Droperidol	144	3.9	3.2–4.5	–	–	–
Midazolam	142	3.8	3.2–4.4	–	–	–
Others	138	3.8		3	0.5	
Total	3,715	100		469	100	

a World Health Organization, Collaborating Centre for Drug Statistics Methodology. Guidelines for ATC classification and DDD assignment 2014. Oslo; 2014 [cited 2016 Mar 15]. Available from: whocc.no/atc_ddd_index/

b Stratification was held only for spinal anesthesia and topical anesthesia. Due to its very low frequency, general anesthesia was considered missing and epidural anesthesia was analyzed together with spinal anesthesia.

The distribution of therapeutic groups according to delivery type (cesarean or vaginal delivery) is found in Table 4. Drugs that act on the nervous system, alimentary tract and metabolism, cardiovascular system and respiratory system, as well as anti-infective agents, were more used in mothers who had cesarean delivery than in those who had vaginal delivery. Greater emphasis should be given to anti-infective agents (1.6 times higher use) and drugs that act on the cardiovascular system and respiratory system, whose prevalence rates were more than double in mothers who had caesarean delivery compared with those who had vaginal delivery. Among the drugs for the nervous system, bupivacaine was 33% more used in mothers who had cesarean delivery and morphine was used only by them, with prevalence of 8.2%. In contrast, paracetamol was two times more used in mothers who had vaginal delivery (12.5%). As for anti-infective agents, cephalotin was the most used (8.3%) and used only in women who had cesarean delivery. Metaraminol was the most used drug that acts on the cardiovascular system (5.1%), used only by women who had cesarean delivery. For vaginal deliveries, compared with cesarean deliveries, the most frequent groups were those that act on the musculoskeletal system (1.4 times higher), systemic hormonal preparations (1.7 times higher), drugs that act on the blood and blood-forming organs (2.5 times higher), and dermatological drugs (1.5 times higher), as shown in Table 4. Ketoprofen was used only in women who had caesarean deliveries (8.6%); however, diclofenac was 2.8 times more used in women who had vaginal delivery (17.0%, compared with 4.5% for cesarean deliveries). As for systemic hormonal preparations, oxytocin is highlighted, used in 8.6% of mothers who had caesarean delivery and in 16.2% of mothers who had vaginal delivery. Ferrous sulfate was three times more used in mothers who had vaginal delivery (14.3%, compared with 3.6% for cesarean deliveries). The prevalence rates of the drugs are not presented in the table.

**Table 4:** Distribution of therapeutic groups used in delivery hospitalization according to the Anatomical Therapeutic Chemical Classification^a^ (ATC; level 1), stratified by delivery type. 2015 Pelotas Births Cohort (June to October), Brazil.

Therapeutic groups^b^		Cesarean delivery			Vaginal delivery		
		n	%	95%CI	n	%	95%CI
N – Nervous System		3,720	32.8	31.9–33.6	554	20.7	19.1–22.2
	N01BB01 – Bupivacaine	1,120	10.4	9.8–11.0	195	7.8	6.7–8.8
	N02AA01 – Morphine	887	8.2	7.7–8.8	3	0.1	-0.01–0.2
	N02BE01 – Paracetamol	446	4.2	3.7–4.5	313	12.5	11.2–13.8
A – Alimentary tract and metabolism		1,631	14.4	13.7–15.0	304	11.4	10.1–12.5
	A04AA01 – Ondansetron	497	4.6	4.2–5.0	6	0.2	0.04–0.4
	A03FA01 – Metoclopramide	202	1.9	1.6–2.1	50	2.0	1.4–2.5
	A04AD51 – Association containing scopolamine	147	1.4	1.1–1.6	93	3.7	2.9–4.4
M – Musculoskeletal system		1,422	12.5	11.9–13.1	466	17.4	15.9–18.9
	M01AE03 – Ketoprofen	916	8.6	8.0–9.0	43	1.7	1.2–2.2
	M01AB05 – Diclofenac	488	4.5	4.1–5.0	423	17.0	15.4–18.4
J – Anti-infectives for systemic use		1,224	10.8	10.2–11.3	137	5.1	4.2–5.9
	J01DB03 – Cephalothin	897	8.3	7.8–8.9	12	0.5	0.2–0.7
	J06BB01 – Anti-D immunoglobulin (Rh)	71	0.7	0.5–0.9	36	1.4	0.9–1.9
H – Systemic Hormonal preparations		1,142	10.1	9.5–10.6	459	17.2	15.7–18.5
	H01BB02 – Oxytocin	924	8.6	8.1–9.1	433	16.2	15.8–18.8
C – Cardiovascular System		897	7.9	7.4–8.4	66	2.5	1.8–3.0
	C01CA09 – Metaraminol	548	5.1	4.7–5.5	0	0	
	C01CA01 – Etilefrine	134	1.3	1.0–1.4	2	0.1	-0.3–1.9
B – Blood and blood-forming organs		551	4.9	4.4–5.2	458	17.1	15.6–18.5
	B03AA07 – Ferrous sulfate	389	3.6	3.2–4.0	357	14.3	12.9–15.6
	B05XA03 – Sodium chloride	126	1.1	0.9–1.3	3	0.1	0.001–0.02
R – Respiratory system		349	3.1	2.8–3.4	22	0.8	0.5–1.1
	R06AA02 – Diphenhydramine	172	1.6	1.3–1.8	11	0.4	0.1–0.7
	R06AD02 – Promethazine	171	1.6	1.3–1.8	11	0.4	0.1–0.7
D – Dermatologicals		220	1.9	1.6–2.1	128	4.8	3.9–5.5
Others		184	1.6		81	3.0	
Total		11,340	100		2,702	100	

a World Health Organization, Collaborating Centre for Drug Statistics Methodology. Guidelines for ATC classification and DDD assignment 2014. Oslo; 2014 [cited 2016 Mar 15]. Available from: whocc.no/atc_ddd_index/

b Of the 14,383 drugs used, 368 were not classified by the ATC system due to absence of reason for use, which may change the classification of the drug.

Table 5 shows the distribution of therapeutic groups according to mother age. As for those that act on the musculoskeletal system and on the blood and blood-forming organs, the greater the age, the lower the prevalence of use, while for those that act on the cardiovascular system, the greater the age, the greater the use. Considering the musculoskeletal system, diclofenac and ketoprofen were the most used drugs; for diclofenac, the younger the mothers, the greater the use, while the opposite occurred with ketoprofen (data not presented in the table). In the therapeutic group of drugs that act on the blood and blood-forming organs, ferrous sulfate was the most used, and the younger the mothers, the greater the use. In the group of drugs that act on the cardiovascular system, metaraminol was the most used drug, but with no significant difference according to age group (data not presented in the table).

**Table 5:** Distribution of most used drugs in delivery hospitalization according to age, according to the Anatomical Therapeutic Chemical (ATC; level 1)* classification system for pharmacological groups. 2015 Pelotas Births Cohort, Brazil.

Pharmacological groups*	13–19 years			20–30 years			31–45 years		
	n	%	95%CI	n	%	95%CI	n	%	95%CI
N – Nervous System	591	29.1	27.2–31.1	2,202	30.2	29.0–31.1	1,481	31.5	30.1–32.8
M – Musculoskeletal system	303	15.0	13.4–16.5	1,006	13.8	12.9–14.5	579	12.3	11.4–13.3
A – Alimentay tract and metabolism	286	14.1	12.6–15.6	997	13.7	12.9–14.5	652	13.9	12.9–14.9
H – Hormonal preparations for systemic use	234	11.5	10.1–12.9	835	11.5	10.7–12.1	532	11.3	10.3–12.2
J – Anti-infective agents for systemic use	177	8.8	7.4–9.9	722	9.9	9.2–10.6	462	9.8	8.9–10.6
B – Blood and blood-forming organs	177	8.8	7.5–9.9	540	7.4	6.8–8.0	292	6.2	5.5–6.9
C – Cardiovascular System	106	5.2	4.2–6.2	481	6.6	6.2–7.3	376	8.0	7.1–8.7
D – Dermatologicals agents	59	2.9	2.1–3.6	175	2.4	2.1–2.8	114	2.4	2.0–2.8
R – Respiratory system	58	2.8	2.2–3.7	183	2.5	2.1–2.9	130	2.8	2.3–3.3
G – Genitourinary system and sex hormones	33	1.6	1.0–2.1	108	1.5	1.2–1.7	54	1.2	0.8–1.4
Others	3	0.2		39	0.5		28	0.6	
Total	2,027	100		7,288	100		4,700	100	

* World Health Organization, Collaborating Centre for Drug Statistics Methodology. Guidelines for ATC classification and DDD assignment 2014. Oslo; 2014 [cited 2016 Mar 15]. Available from: whocc.no/atc_ddd_index/

## DISCUSSION

This study highlights the high drug use in mothers in the perinatal period, mainly in cesarean deliveries, and the prevalence of drugs that act on the nervous system. A cross-sectional study carried out in four maternity hospitals of Belo Horizonte also reported extensive use of drugs in the immediate postpartum period, with 96% of women being given some form of drug^1^. Another study, carried out with 24 recent mothers in a maternity hospital of Ceará, showed the use of drugs in the immediate postpartum period in 63% of recent mothers and associated this use to cesarean delivery^11^.

The mean number of drugs used was higher in SUS hospitalizations, both in prepartum/during delivery and postpartum periods, inconsistently with the findings of Perini et al.^8^, which showed higher mean number of drugs in private hospitals in both periods. It is important to consider that the mean number of drugs in our study was higher in medical school hospitals, whose hospitalizations are mainly by the SUS. Drug use in these hospitals is higher probably because of the complexity of care, since they are the only ones in the city that have NICU. On the other hand, analysis of delivery type in relation to hospital type shows that the exclusively SUS medical school hospital conducts more vaginal deliveries than cesarean deliveries, while exclusively private and health insurance hospitals conduct more cesarean deliveries than vaginal deliveries. This reflects reality, because women who have health insurance or who intend to have private delivery usually schedule it to the most convenient date.

Cesarean delivery and epidural anesthesia are pointed out as the main factors associated with high consumption of drugs in the perinatal period^8^ ^,^ ^11^, which was also observed in our study, especially for drugs that act on the cardiovascular and respiratory systems, showing higher exposure to drugs and consequently higher risk to the safety of the mother and the baby. Metaraminol was only used in women who underwent caesarean delivery and, consequently, received spinal anesthesia. Metaraminol is a sympathomimetic agent used in the prophylaxis and treatment of hypotension in women who have cesarean delivery^17^.

On the other hand, women who had vaginal delivery were given more drugs that act on the blood and hematopoietic system than those who had cesarean delivery. Ferrous sulfate was the most used drug in this class, especially by younger mothers, who have a higher risk of developing anemia in gestation^18^, because they are in growth phase^19^. Dermatological drugs and those that act on the musculoskeletal system and hormonal preparations, which are generally safer in the perinatal period, due to presenting lower risk of serious adverse reactions and to being eliminated in breast milk^20^ ^,^ ^21^, were also among the most used when vaginal delivery was conducted. Thus, the drugs most used in cesarean delivery, such as bupivacaine and morphine, represent higher risk for the mother and the baby^3^ ^,^ ^22^, which points to one more harmful exposure in cesarean delivery.

A concerning fact as to the use of drugs in the perinatal period is that mothers are initiating breastfeeding, and they can interfere with milk production or cause unwanted effects on babies. Bupivacaine, medicine of the nervous system most used in this study, can interfere both beneficially and deleteriously with breastfeeding; however, there is lack of evidence, because the results are very inconsistent due to the via and form of administration, doses, time of use, etc. For example, in a randomized clinical trial conducted by Jolly et al., additional use of bupivacaine caused greater comfort during breastfeeding^23^. The use of morphine, in turn, can delay the initiation of breastfeeding and even cause child drowsiness, central nervous system depression, and even death. Even so, if the use of opiates is necessary for pain control, morphine in low doses is preferable in relation to the others^24^. Newborns seem to be particularly sensitive to the effects of narcotic analgesics, even in small doses^25^.

There are reports of episodes of cyanosis in the baby after the use of dipyrone (metamizole) by the breastfeeding mother. Dipyrone (metamizole) and its metabolites reach high concentrations in breast milk, remaining for up to 48 hours. It is recommended the use of other non-steroidal analgesic that is safer, or that the mother does not breastfeed for 48 hours after using the drug^26^ ^,^ ^27^.

Oxytocin appears as a medication widely used in this study, being widely used during childbirth. It is a hormone released during breastfeeding that seems to have tranquilizing effect on the mother^20^; however, the administration of exogenous oxytocin to mothers with difficulty in breast-feeding showed no beneficial effect on the success of lactation or on the treatment of breast engorgement. It seems to have no effect on the child. On the other hand, several studies suggest that oxytocin administered during delivery can negatively affect breastfeeding, possibly reducing the suction behavior in the newborn^28^ ^–^ ^30^. A study pointed out that all rhythmic reflexes, the antigravity reflex, and the primitive neonatal reflexes were inhibited by the administration of oxytocin during delivery, an effect unrelated to dose, which could also detrimental to breastfeeding^31^ ^,^ ^32^.

Among the medicines that act on the musculoskeletal system, diclofenac was the most widely used. Despite being widely used as post-cesarean delivery analgesic, there are few data on the excretion of this drug in breast milk, with most authors considering its use acceptable during breastfeeding^33^. Diclofenac has proven effective to reduce the dose of opioids in postpartum analgesia, with no important adverse reactions, such as bleeding or uterine atonia^33^.

The longer the length of hospital stay, the greater was the number of drugs used. This leads us to think that mothers who were hospitalized more days probably had postpartum complications or are women with more co-morbidities, requiring more monitoring. On the other hand, it was observed that a significant number of women (15%) had only one day of hospitalization. According to the Brazilian Ministry of Health (MS), there is no official definition about postpartum length of hospital stay, with only one reference in Ordinance 1,016 noting that discharges should not be given before 48 hours^34^. The American Academy of Pediatrics (AAP), and the American College of Obstetrics and Gynecology (ACOG) define as early discharge that which occurs in the first 48 hours after delivery and as very early discharge that which occurs within the first 24 hours. They recommend 48 hours as mean time of hospitalization for non-complicated vaginal delivery and 96 hours for cesarean delivery^35^. This study found that 35% of mothers had early discharge and 15% had very early discharge, which puts at risk the health of both the mother and the baby. Analysis of hospitalization days in relation to hospitalization type (SUS, private health care, or health insurance) shows that women who had private or health insurance hospitalization stayed fewer days in the hospital than women who had SUS hospitalization, which reflects the following of the guidelines of the Ministry of Health by public hospitals and not by private hospitals.

A theoretical limitation of the study could be its conduct period, from June to October, very cold months in the city. However, a previous study^8^ showed no seasonality in the use of medicines in the perinatal period and highlights that it enables conducting such researches in short periods of time, saving time and money. Another limitation could be that the sample was a sub-sample of the 2015 Pelotas births cohort, which could have different characteristics. However, after analysis (not shown in the results because it was performed only for comparative purposes) it was shown that the sociodemographic characteristics of the mothers participating in this study are similar to those of the other mothers of the cohort.

This study found high drug consumption in the delivery hospitalization period, and the main factors were cesarean delivery and epidural anesthesia. The most used therapeutic group was that of drugs that act on the nervous system.
